# Using a Systems Wide Approach to Improve Medical Emergencies in a High Secure Forensic Psychiatry Setting

**DOI:** 10.1192/bjo.2024.394

**Published:** 2024-08-01

**Authors:** Brooke Marron, Cormac Maguire

**Affiliations:** The State Hospital, Carstairs, United Kingdom

## Abstract

**Aims:**

Patients with severe mental illness are at a significantly higher risk of poor physical health outcomes than the general population and die on average 15–20 years earlier. The Royal College of Psychiatrists has published extensively on how to improve routine physical health monitoring in this cohort. Despite this, there is little data or guidance on improving emergency medical care for this cohort. We aimed to analyse and optimise the process of patients being sent to general hospital on an emergency basis.

**Methods:**

A review was undertaken of the clinical notes for medical emergencies over a 12 month period by two core psychiatry trainees. Site visits and interviews with local A&E department clinicians, the covering General Practitioner and pharmacy were completed. A questionnaire was distributed to all nursing staff to gather their perspective on the considerations for emergency medical transfers out of The State Hospital.

**Results:**

On review of the case notes, 44/44 emergency outings were deemed to be clinically necessary for investigations/interventions that would not have been possible on-site. Qualitative methodology highlighted a disconnect amongst staff groups and stake holders regarding thresholds for transfers relating to medical emergencies leading to a high level of staff dissatisfaction.

**Conclusion:**

The interface between psychiatric and medical services is an area of risk to patients. Levels of staff confidence, knowledge and available resources all contribute to the risk of transfer. Further work is required to explore other aspects of patient care and treatment which can be impacted as a consequence of emergency transfers.

